# Red Wine as an Aromatase Inhibitor: A Narrative Review

**DOI:** 10.7759/cureus.59587

**Published:** 2024-05-03

**Authors:** Joseph Pergolizzi Jr, Jo Ann K LeQuang, Morgan Wagner, Rania Salah, Peter Magnusson, Giustino Varrassi

**Affiliations:** 1 Pain Management, NEMA Research, Inc., Naples, USA; 2 Scientific Communications, NEMA Research, Inc., Naples, USA; 3 Entrepreneur Program, NEMA Research, Inc., Naples, USA; 4 Medical School, Alfaisal University College of Medicine, Riyadh, SAU; 5 School of Medical Sciences, Örebro University, Örebro, SWE; 6 Pain Medicine, Paolo Procacci Foundation, Rome, ITA

**Keywords:** postmenopausal breast cancer, breast cancer care, breast cancer, aromatase inhibitor therapy, red wine

## Abstract

As estrogen-dependent breast cancer is more affected by the local production of estrogen via aromatase than serum estrogen, aromatase inhibitors for treating breast carcinomas in postmenopausal women have been developed. As the aromatase enzyme converts endogenous androgen to estrogenic compounds, its blockade lowers the in situ production of estrogen, demonstrated to encourage tumor proliferation. Red wine, but not white wine, may have aromatase-inhibiting properties that are being elucidated, although the exact mechanisms of action are not known. Polyphenols, tannins, and resveratrol have all been implicated as aromatase blockers, and there may also be synergistic interplay among selected constituents. The role of red wine would be in chemoprevention, the use of natural or synthetic substances to retard, block, or reverse cancer. One gene encodes aromatase, so aromatase inhibition would stop endogenous estrogen production. The role of aromatase inhibition in breast cancer in premenopausal women is not clear. While animal studies have demonstrated that red wine contains constituents that could block aromatase in vivo, the benefits also exist with nonalcoholic grape seed extract. Further investigation is needed but there are challenges in designing appropriate clinical trials for a substance as variable as red wine. While there is insufficient evidence to advocate for red wine as an aromatase inhibitor, there is sufficient evidence to warrant further investigation.

## Introduction and background

Postmenopausal breast cancer is often an estrogen-dependent carcinoma [1]. Estrogen can be produced in situ by the actions of the aromatase cytochrome P450-19 enzyme that synthesizes C19 androgens into aromatic C18 estrogenic steroids [2-4]. The most powerful endogenous estrogen is estradiol, synthesized by the action of aromatase [5]. Local estrogen plays a greater role in tumor growth than serum estrogen [6-8]. Tumors of the breast exhibit upregulated aromatase compared to noncancer tissue [4]. Thus, the treatment strategy of aromatase inhibition was developed based on the premise that suppressing in situ estrogen synthesis would offer cancer protection [5,8].

Chemoprevention is evolving new anticancer techniques deploying natural or synthetic substances to block, delay, or reverse carcinogenesis [9]. Pharmacologic aromatase inhibitors are prescribed for postmenopausal breast cancer patients but are associated with potential side effects such as joint problems and vasomotor symptoms. Prolonged exposure to aromatase-inhibiting drugs may elevate the patient’s risk for cardiovascular events as well as fractures [10]. The use of red wine is currently being explored as a possible chemopreventive substance. While alcohol consumption is associated with an increased risk for breast cancer, wine is a complex substance with multiple nutritional constituents that may act alone or in combination [8]. Red wine contains specific phytochemicals not found in white wine, and these phytochemicals are associated with aromatase inhibition [11].

The purpose of this narrative review was to consider the current literature on the potential activity of red wine in aromatase inhibition and its possible role as a chemopreventive substance.

## Review

Methodology

In November 2023, the PubMed database was searched for the keywords “red wine aromatase inhibitor” with no delimiters (11 results), “wine aromatase inhibitor” with no delimiters (11 results), and “wine aromatase” (17 results). PubMed was also searched for “red wine breast cancer” (two results), “wine breast cancer” (five results), and “wine cancer prevention” (15 results). There was only one randomized clinical trial related to this subject. Google Scholar was also searched for the same keywords. We also searched the bibliographies of relevant articles. As this is an emerging topic with limited literature and a paucity of studies, many articles found provided background rather than specific information on our research topic.

Red wine

Wine has been a part of civilization for millennia in most parts of the world and plays a dietary, social, and even religious role in many cultures [12]. The medical study of wine is complicated by the many varieties and enological techniques, soils, grape varieties, and other factors that account for so many distinctive vintages. It is scientifically difficult to talk about wine or even red wine globally because specific types, vintages, or grape varieties offer different health benefits.

Both red and white wine contain comparable amounts of alcohol, but red wine contains more polyphenols than white wine. In producing red wine, the skins and seeds of grapes are not removed, which elevates its polyphenolic content [8]. The potential aromatase-inhibiting properties of red wine have been presumed related to the unique phytochemicals absent in white wine [11]. These naturally occurring aromatase inhibitors also occur in grapes, grape skins, and grape extract [13].

There are several constituents in red wine synthesized along a common pathway from phenylalanine via polyketide condensation reactions. Resveratrol synthase and chalcone synthase are precursors of acyl-CoA derivatives, which metabolize fatty acids. Polymeric aggregation produces viniferins (antifungals) and procyanidins (antioxidants that also inhibit platelet aggregation) [12]. The potential synergistic interactions among the constituents have not been well studied but may play a role [14]. The main constituents of red wine are described in Table 1.

**Table 1:** Red wine and its constituents. Red wine contains 800 or more individual chemical compounds and the exact composition varies by type of wine, grape, vintage, and other factors. Red wine contains <1% polyphenols, although these substances play a major role in wine’s health benefits [11-14].

Name	Description	Characteristics
Phenolic acids		
P-coumaric	One of three isomers of hydroxycinnamic acid	More soluble in ethanol than in water
Cinnamic	Unsaturated carboxylic acid exists as a cis- and trans-isomer	More soluble in ethanol than water. Also found in the bark of Cinnamomum trees
Caffeic	Hydroxycinnamic acid with phenolic and acrylic functional groups	Antioxidant, anti-inflammatory, and anticancer. Also found in coffee, turmeric, and other plants
Gentisic	Dihydroxybenzoic acid	Plausible anti-inflammatory and neuroprotective effects
Ferulic	Hydroxycinnamic acid	Phenolic phytochemical. Antioxidant and plausible antiaging properties
Vanillic	4-hydroxy-3-methoxybenzoic acid which is an oxidized form of vanillin	Antioxidant and anti-inflammatory
Flavan-3-ols		
Catechin	Natural phenol produced as a secondary metabolite	Promotes nitric oxide production via the vascular endothelium, inhibits thromboxane synthesis in platelets and leukotriene in neutrophils, arrests tumor growth, and inhibits carcinogenesis in experimental models. Has antioxidant properties
Epicatechin	Polyphenol flavonoid	Found in green tea, apples, and berries, and is associated with improved cognition
Myricetin	A polyphenol, 3,3’,4’,5,5’7-hexahydroxyflavone	Found in fruit, nuts, berries, and tomatoes. Structurally similar to quercetin. Has antioxidant and anticancer properties
Quercetin	Polyphenol flavonoid, plant pigment	Promotes nitric oxide production via the vascular endothelium, inhibits thromboxane synthesis in platelets and leukotriene in neutrophils, arrests tumor growth, and inhibits carcinogenesis in experimental models. Has antioxidant and anti-inflammatory properties
Tannins		
Tannins	Polymers, often consisting of long strings of condensed flavan-3-ol molecules	Tannin concentration varies by wine. Tannin may affect serotonin levels and has been implicated in “red wine headaches”
Trihydroxy stilbenes		
Resveratrol	A stilbenoid or natural phenol produced by the plant under stress, attack by bacteria or fungi, or another type of injury. Found in the skin of grapes	Promotes nitric oxide production via the vascular endothelium, inhibits thromboxane synthesis in platelets and leukotriene in neutrophils, arrests tumor growth, and inhibits carcinogenesis in experimental models. One of the most interesting constituents of red wine in terms of health benefits
Polydatin (piceid)	3,4’,5-trihydroxystilbene-3-β-D-glucoside	Found in peanuts, hops, and cocoa. Beneficial effects on vascular endothelial cells

While hundreds of compounds have been identified in red wine, its two major bioactive compounds are polyphenols and ethanol [15]. Although resveratrol is perhaps the best known of these bioactive polyphenols, red wine contains about 30 times the amount of flavonols as resveratrol. The main flavonols include 3,3’,4’,5,5’7-hexahydroxyflavone (myricetin) and 3,3’,4’,5,7-pentahydroxyflavone (quercetin). These two compounds can comprise half of all flavonol content in red wine. Myricetin is anticarcinogenic and antioxidant [16] and has been shown to reduce cancer development in mice [17]. Resveratrol, a prominent constituent in red wine, has been implicated as an aromatase inhibitor. Structurally similar to estrogen, resveratrol has agonist and antagonist activity at resveratrol receptors [18].

Carcinogenesis and chemoprevention

Carcinogenesis involves a methodical series of distinct molecular steps, typically over a prolonged period, resulting in changes to cellular structures. These interlocking steps can be broadly grouped into the following three phases: initiation, promotion, and progression [19]. Breast cancer may take years to advance from initiation to malignancy, and it is in this latency period that opportunities may exist for chemopreventive strategies such as aromatase inhibition [9].

Chemoprevention utilizes natural and/or synthetic substances to slow down, block, or reverse carcinogenesis [20]. The localized or in situ synthesis of estrogen promotes tumor growth in breast cancer to a greater extent than the circulating estrogen levels [11]. In a study using ultraviolet absorbance analysis, high-performance liquid chromatography profiling, and accurate mass-mass spectrometry along with nanospray tandem-mass spectrometry, most of the beneficial compounds identified in red wine for reducing estrogen synthesis were procyanidin B dimers and aromatase inhibitors. The most powerful procyanidin B dimer competes with the binding of the androgen substrate. Procyanidin B dimers can inhibit the growth of androgen-related tumors, which implies that they are aromatase inhibitors that reduce estrogen in situ [11]. Moreover, grape seed extract contains procyanidin dimers that have been shown to be in vitro aromatase inhibitors. In a mouse study, grape seed extract functioned in a dose-dependent manner to inhibit tumor growth [21].

Aromatase inhibition

Estrogen has been implicated in both carcinogenesis and breast cancer progression, making estrogen deprivation an appropriate objective in new drug development to treat or prevent breast cancer and cancer recurrence [22]. While synthetic aromatase inhibitors, which block the conversion of endogenous androgens to estrogenic compounds, have been evaluated in breast cancer patients, less is known about naturally occurring substances that may exert similar effects [23]. The lowering of estrogen synthesis in the body is viewed as both beneficial and chemopreventive for patients with hormone-related breast cancer. Although red wine appears to inhibit aromatase, the mechanisms remain unknown [23].

The cytochrome P450-19 enzyme is aromatase. It is the rate-determining step for estrogen synthesis from androgen. The transcriptional control of the *CYP19* gene is specific for different types of cells, that is, Promoter 1.3/II is used most frequently for breast cancer cells. A positive feedback loop for the synthesis of estrogen was recently identified as estrogen receptor (ER) alpha-expressing SK-BR-3 cells. Resveratrol reduced the estradiol-induced quantity of mRNA in the SK-BR-3 cells; the SK-BR-3 cells express ER alpha. Resveratrol limited the nongenomic induction of estrogen on CYP19 [24]. As a single gene encodes the enzyme that produces estrogen in the body, inhibiting this enzyme is tantamount to stopping the endogenous synthesis of estrogen altogether. Aromatase inhibition has been used in the treatment of both breast cancer and endometriosis, but it is not clear if aromatase blockade will have the same effect on endometrial cancer or uterine fibroids [25]. When aromatase is overexpressed in pathologic tissue, cellular composition changes to favor cells that promote breast cancer, molecular alternations in stromal cells bind transcription enhancers rather than inhibitors and initiate transcription, and heterozygous mutations can occur which leads to estrogen formation [25].

Aromatase expression appears to increase with body weight and older age [26]. For postmenopausal breast cancer patients, aromatase inhibition has emerged as a cornerstone of managing and treating ER-positive breast cancer, but the role of aromatase inhibition in premenopausal women with breast cancer remains unclear. In a healthy woman, the aromatase enzyme facilitates the conversion of androstenedione and testosterone into estrogen. A pharmacologic aromatase inhibitor blocks this activity and results in increases in serum blood T as well as concomitant drops in estradiol, estrone, and sex-hormone-binding globulin levels [22]. Thus, the paradigm for estrogen depletion is to inhibit its biosynthesis in the body by interrupting the final stage in the synthetic sequence (androgens > estrogen). This has led to the development of several aromatase-inhibiting drugs, such as tamoxifen, anastrozole, exemestane, and letrozole [27]. Of these agents, letrozole may be the most potent [28] and has the most direct evidence for use in patients who require estrogen-deprivation treatments [29]. However, as these drugs are associated with significant treatment-limiting side effects, if a natural alternative is safe, effective, and available, it would clearly offer an advantage [30].

Red wine in the context of cancer care

Alcohol is considered a risk factor for breast cancer, although the exact mechanisms are not known. While both red and white wine contain alcohol, red wine does not pose as much of a risk for breast cancer patients as white wine [31,32]. For women, alcohol intake has been associated with breast cancer and several other cancers, including oral cancer, rectal cancer, larynx cancer, and liver cancer [32]. In a meta-analysis of six prospective studies (n = 322,647 followed for up to 11 years) in which each study presented a minimum of 200 incident cases of breast cancer, investigators found that breast cancer risk increased linearly with the volume of alcohol consumed, but the specific type of alcoholic beverage did not play a significant role [33]. A prospective observational study (n = 105,986) following women from 1980 until 2008 found that cumulative alcohol intake over the adult years was associated with an increased risk of breast cancer, particularly alcohol consumption early and/or late in life. Binge drinking was associated with elevated breast cancer risk, but not frequency of alcohol intake [34].

Consumption of alcohol increases hepatic aromatase activity, raising plasma levels of estradiol and decreasing serum testosterone. When de-alcoholized wines were tested by lyophilizing the wine and reconstituting it using water to the original volume, the effect of alcohol on aromatase was demonstrated to be insignificant [8]. In fact, women who consume one alcoholic drink daily have a 12% higher risk of breast cancer than women who do not drink [32,35,36]. However, these studies treated all alcoholic beverages as equivalents and did not differentiate between red and white wine or compare red to white wine; thus, it is possible that red wine had a beneficial effect that was masked by this study design [31].

It has been stated that the biological composition of red wine offers greater cancer protection in humans than many other dietary sources, including fruits, vegetables, and other so-called “superfoods” [12]. The constituents in red wine are synthesized by polyketide condensation reactions along a pathway from phenylalanine. In this process, the phenolic acids, trihydroxy stilbenes, and flavonoids are synthesized. Resveratrol and chalcone synthases may be considered the precursors of acyl-CoA derivatives. Competitive interaction between resveratrol synthase and chalcone synthase helps regulate metabolism. Polymeric aggregation then produces long-chain viniferins, with antifungal properties, and procyanidins, which are antioxidants that can inhibit platelet aggregation [12]. Catechin, quercetin, and resveratrol encourage the vascular endothelium to produce nitric oxide, which, in turn, inhibits thromboxane synthesis in platelets and leukotriene in neutrophils. Because it can regulate the synthesis and secretion of lipoproteins, it can slow tumor growth and inhibit carcinogenesis [12].

Damage to vascular walls and DNA may be caused by inducible nitric oxide synthase (iNOS), which occurs in humans with atherosclerosis and tumor genesis. Quercetin and resveratrol at micro-molar ranges were able to suppress iNOS gene expression and reduce nitric oxide production. Further, polyphenols scavenged nitric oxide under physiologic conditions [37]. While ethanol did not reduce iNOS or nitric oxide production, when it was present in the range of 0.1% to 0.75%, it enhanced the activity of grape polyphenols in a concentration-dependent manner [37].

Preclinical and clinical studies

Grape seed extract contains procyanidin dimers that have been shown to be in vitro aromatase inhibitors. In a mouse study, grape seed extract functioned in a dose-dependent manner to inhibit tumor growth [21]. In a preclinical study, a 20% acetonitrile fraction extract was administered by gavage to mice and eliminated aromatase-induced hyperplasia and mammary tissue changes attributed to aromatase [8]. The compounds associated with aromatase inhibition were procyanidin B-dimers, which in murine studies reduced androgen-induced tumor growth, implying that they could suppress estrogen synthesis [11]. An aromatase assay was conducted using human placental microsomes. In total, 11 red and four white wines were evaluated. The white wines did not suppress aromatase, but all of the red wines did. This evaluation suggests that alcohol is not involved in aromatase inhibition, but rather the aromatase inhibitory effect relies on one or more constituents found in red, but not white, wine [11].

To our knowledge, there is only one controlled clinical study in which red wine was examined as a potential nutritional aromatase inhibitor. In a cross-over study with 36 healthy premenopausal participants (mean age: 36 years), the first group drank 8 ounces of red wine (Cabernet Sauvignon, BV Coastal 2003) daily for a month, and then, in the second month, they drank white wine (Chardonnay, BV Coastal, 2003). There were two study arms and the second arm did the two phases in reverse. Twice during the menstrual cycle, blood was tested for estradiol, estrone, androstenedione, total and free testosterone levels, sex hormone-binding globulin, luteinizing hormone, and follicle-stimulating hormone. The red wine groups had significantly higher free testosterone levels and significantly lower sex hormone-binding globulin levels. Estrione levels were lower in the red wine group but the difference did not achieve statistical significance. Investigators concluded these results suggested that red wine acted as an aromatase inhibitor in these women, aligning with the observation that red wine does not seem to increase breast cancer risk [31].

Discussion

Red wine is an ancient and important part of global culture and health, but it has posed methodology challenges in terms of clinical investigation. The many types and varieties of red wine make it difficult to talk about red wine generically, much less compare doses or even constituents. While it appears that red wine, but not white wine, may be a natural aromatase inhibitor, which, in turn, may confer benefits on postmenopausal women with breast cancer, the clinical evidence we have to date is limited and not likely to expand in the near term. There is not sufficient evidence to state that red wine is a safe, effective treatment for breast cancer in postmenopausal women or that it is a safe and effective chemopreventive dietary product in humans [38]. However, it would be fair to say that the current evidence warrants further and deeper investigation [23].

The primary challenge in this type of research is that substances such as wine cannot be evaluated in the same manner as manufactured synthetic drugs administered with carefully controlled doses according to a clinical protocol. Red wine is not the same, and it may be that certain wines are more beneficial than others. It is not known how to stratify them. Second, red wine contains alcohol, meaning that it would be possible to consume quantities large enough to negate any potential benefits. Finally, the mechanisms of action involved have been implicated, and, although they are intriguing, they are not yet clear. Designing clinical studies for this type of research poses interesting and unusual challenges.

Our article has several limitations. This is a narrative review of a topic on which a very limited amount of literature exists. There is a paucity of scientific studies to analyze. Further, there are cultural and scientific biases that come into play, particularly among the lay public that may favor ancient natural products as remedies for modern disease, and scientific biases among investigators, who find serious analysis of such products speculative and unproductive. Based on the current literature, it is not advisable to use red wine as a chemopreventive agent or a cancer-fighting nutritional supplement, but there is sufficient evidence to investigate it further concerning its ability to inhibit the aromatase enzyme.

## Conclusions

Aromatase inhibition is an important treatment strategy for estrogen-dependent breast cancer in postmenopausal women, and red wine may offer a natural form of aromatase blockade. This benefit occurs in red but not white wine, suggesting that it involves some of the specific polyphenols and other constituents of red wine, which is produced using grape skins and pits as well as the fruit. Aromatase is an enzyme that synthesizes androgen into estrogenic compounds. This aromatase-synthesized localized estrogen can be far more damaging to malignancies than circulating estrogen. A few preclinical studies demonstrate that red wine can block aromatase in vivo, and benefits are present with red wine extracts as well as red wine-containing alcohol. Although there are few clinical trials in humans, one found that red wine drinkers had higher free testosterone levels and low sex hormone-binding globulin levels than white wine drinkers. While further investigation is warranted, the evidence does not yet support the use of red wine as an aromatase inhibitor for breast cancer patients.

## References

[1] Nicolini A, Ferrari P, Duffy MJ (2018). Prognostic and predictive biomarkers in breast cancer: past, present and future. Semin Cancer Biol.

[2] Esteban JM, Warsi Z, Haniu M, Hall P, Shively JE, Chen S (1992). Detection of intratumoral aromatase in breast carcinomas. An immunohistochemical study with clinicopathologic correlation. Am J Pathol.

[3] Bulun SE, Price TM, Aitken J, Mahendroo MS, Simpson ER (1993). A link between breast cancer and local estrogen biosynthesis suggested by quantification of breast adipose tissue aromatase cytochrome P450 transcripts using competitive polymerase chain reaction after reverse transcription. J Clin Endocrinol Metab.

[4] Lu Q, Nakmura J, Savinov A (1996). Expression of aromatase protein and messenger ribonucleic acid in tumor epithelial cells and evidence of functional significance of locally produced estrogen in human breast cancers. Endocrinology.

[5] Brueggemeier RW (2002). Aromatase inhibitors in breast cancer therapy. Expert Rev Anticancer Ther.

[6] Tekmal RR, Ramachandra N, Gubba S (1996). Overexpression of int-5/aromatase in mammary glands of transgenic mice results in the induction of hyperplasia and nuclear abnormalities. Cancer Res.

[7] Yue W, Wang JP, Hamilton CJ, Demers LM, Santen RJ (1998). In situ aromatization enhances breast tumor estradiol levels and cellular proliferation. Cancer Res.

[8] Eng ET, Williams D, Mandava U, Kirma N, Tekmal RR, Chen S (2002). Anti-aromatase chemicals in red wine. Ann N Y Acad Sci.

[9] He S, Sun C, Pan Y (2008). Red wine polyphenols for cancer prevention. Int J Mol Sci.

[10] Lintermans A, Neven P (2015). Safety of aromatase inhibitor therapy in breast cancer. Expert Opin Drug Saf.

[11] Eng ET, Ye J, Williams D (2003). Suppression of estrogen biosynthesis by procyanidin dimers in red wine and grape seeds. Cancer Res.

[12] Soleas GJ, Diamandis EP, Goldberg DM (1997). Wine as a biological fluid: history, production, and role in disease prevention. J Clin Lab Anal.

[13] Eng ET, Williams D, Mandava U, Kirma N, Tekmal RR, Chen S (2001). Suppression of aromatase (estrogen synthetase) by red wine phytochemicals. Breast Cancer Res Treat.

[14] Bradlow HL, Telang NT, Sepkovic DW, Osborne MP (1999). Phytochemicals as modulators of cancer risk. Adv Exp Med Biol.

[15] Finicelli M, Squillaro T, Di Cristo F, Di Salle A, Melone MA, Galderisi U, Peluso G (2019). Metabolic syndrome, Mediterranean diet, and polyphenols: evidence and perspectives. J Cell Physiol.

[16] Ong KC, Khoo HE (1997). Biological effects of myricetin. Gen Pharmacol.

[17] Mukhtar H, Das M, Khan WA, Wang ZY, Bik DP, Bickers DR (1988). Exceptional activity of tannic acid among naturally occurring plant phenols in protecting against 7,12-dimethylbenz(a)anthracene-, benzo(a)pyrene-, 3-methylcholanthrene-, and N-methyl-N-nitrosourea-induced skin tumorigenesis in mice. Cancer Res.

[18] Wang Y, Lee KW, Chan FL, Chen S, Leung LK (2006). The red wine polyphenol resveratrol displays bilevel inhibition on aromatase in breast cancer cells. Toxicol Sci.

[19] Kundu JK, Surh YJ (2004). Molecular basis of chemoprevention by resveratrol: NF-kappaB and AP-1 as potential targets. Mutat Res.

[20] Surh YJ (2003). Cancer chemoprevention with dietary phytochemicals. Nat Rev Cancer.

[21] Kijima I, Phung S, Hur G, Kwok SL, Chen S (2006). Grape seed extract is an aromatase inhibitor and a suppressor of aromatase expression. Cancer Res.

[22] Miller WR (2004). Biological rationale for endocrine therapy in breast cancer. Best Pract Res Clin Endocrinol Metab.

[23] Singh S (2023). Review on natural agents as aromatase inhibitors: management of breast cancer [in press]. Comb Chem High Throughput Screen.

[24] Wang Y, Ye L, Leung LK (2008). A positive feedback pathway of estrogen biosynthesis in breast cancer cells is contained by resveratrol. Toxicology.

[25] Bulun SE, Lin Z, Imir G (2005). Regulation of aromatase expression in estrogen-responsive breast and uterine disease: from bench to treatment. Pharmacol Rev.

[26] Bulun SE, Zeitoun K, Sasano H, Simpson ER (1999). Aromatase in aging women. Semin Reprod Endocrinol.

[27] Rugo HS (2008). The breast cancer continuum in hormone-receptor-positive breast cancer in postmenopausal women: evolving management options focusing on aromatase inhibitors. Ann Oncol.

[28] Miller WR, Anderson TJ, Dixon JM (2002). Anti-tumor effects of letrozole. Cancer Invest.

[29] Berry J (2005). Are all aromatase inhibitors the same? A review of controlled clinical trials in breast cancer. Clin Ther.

[30] Mukherjee AG, Wanjari UR, Nagarajan D (2022). Letrozole: pharmacology, toxicity and potential therapeutic effects. Life Sci.

[31] Shufelt C, Merz CN, Yang Y (2012). Red versus white wine as a nutritional aromatase inhibitor in premenopausal women: a pilot study. J Womens Health (Larchmt).

[32] Allen NE, Beral V, Casabonne D, Kan SW, Reeves GK, Brown A, Green J (2009). Moderate alcohol intake and cancer incidence in women. J Natl Cancer Inst.

[33] Smith-Warner SA, Spiegelman D, Yaun SS (1998). Alcohol and breast cancer in women: a pooled analysis of cohort studies. JAMA.

[34] Chen WY, Rosner B, Hankinson SE, Colditz GA, Willett WC (2011). Moderate alcohol consumption during adult life, drinking patterns, and breast cancer risk. JAMA.

[35] Ellison RC, Zhang Y, McLennan CE, Rothman KJ (2001). Exploring the relation of alcohol consumption to risk of breast cancer. Am J Epidemiol.

[36] Key J, Hodgson S, Omar RZ (2006). Meta-analysis of studies of alcohol and breast cancer with consideration of the methodological issues. Cancer Causes Control.

[37] Chan MM, Mattiacci JA, Hwang HS, Shah A, Fong D (2000). Synergy between ethanol and grape polyphenols, quercetin, and resveratrol, in the inhibition of the inducible nitric oxide synthase pathway. Biochem Pharmacol.

[38] Lachenmeier DW, Rehm J (2012). Moderate red wine drinking does not help cut women's breast cancer risk. J Womens Health (Larchmt).

